# Plant history and soil history jointly influence the selection environment for plant species in a long‐term grassland biodiversity experiment

**DOI:** 10.1002/ece3.7647

**Published:** 2021-05-11

**Authors:** Peter Dietrich, Nico Eisenhauer, Peter Otto, Christiane Roscher

**Affiliations:** ^1^ Department of Physiological Diversity UFZ Helmholtz Centre for Environmental Research Leipzig Germany; ^2^ German Centre of Integrative Biodiversity Research (iDiv) Halle‐Jena‐Leipzig Leipzig Germany; ^3^ Department of Experimental Interaction Ecology Institute of Biology Leipzig University Leipzig Germany; ^4^ Institute of Biology Herbarium Universitatis Lipsiensis (LZ) Leipzig University Leipzig Germany

**Keywords:** diversity–productivity relationship, eco‐evolutionary feedback, micro‐evolution, plant–soil feedback, transplant experiment

## Abstract

Long‐term biodiversity experiments have shown increasing strengths of biodiversity effects on plant productivity over time. However, little is known about rapid evolutionary processes in response to plant community diversity, which could contribute to explaining the strengthening positive relationship. To address this issue, we performed a transplant experiment with offspring of seeds collected from four grass species in a 14‐year‐old biodiversity experiment (Jena Experiment). We used two‐ and six‐species communities and removed the vegetation of the study plots to exclude plant–plant interactions. In a reciprocal design, we transplanted five “home” phytometers (same origin and actual environment), five “away‐same” phytometers (same species richness of origin and actual environment, but different plant composition), and five “away‐different” phytometers (different species richness of origin and actual environment) of the same species in the study plots. In the establishment year, plants transplanted in home soil produced more shoots than plants in away soil indicating that plant populations at low and high diversity developed differently over time depending on their associated soil community and/or conditions. In the second year, offspring of individuals selected at high diversity generally had a higher performance (biomass production and fitness) than offspring of individuals selected at low diversity, regardless of the transplant environment. This suggests that plants at low and high diversity showed rapid evolutionary responses measurable in their phenotype. Our findings provide first empirical evidence that loss of productivity at low diversity is not only caused by changes in abiotic and biotic conditions but also that plants respond to this by a change in their micro‐evolution. Thus, we conclude that eco‐evolutionary feedbacks of plants at low and high diversity are critical to fully understand why the positive influence of diversity on plant productivity is strengthening through time.

## Graphical abstract

We performed a transplant experiment with offspring of seeds collected in 14‐year‐old plant communities differing in plant species richness. Our findings provide evidence that low and high plant diversity and associated differences in soil community and/or conditions differentially influence eco‐evolutionary feedbacks of plants.

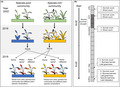

## 1 INTRODUCTION

Long‐term biodiversity experiments have shown a positive relationship between plant diversity and productivity, and that this positive relationship strengthens over time (Cardinale et al., 2007; Fargione et al., 2007; Guerrero‐Ramirez et al., 2017; Reich et al., 2012; Tilman et al., 2006). While the positive effect of plant diversity and productivity is well studied, the reasons for strengthening biodiversity–ecosystem functioning relationships over time are not yet fully understood (Eisenhauer et al., 2019; Guerrero‐Ramirez et al., 2017; Reich et al., 2012). Two processes are suspected to be the main drivers for the positive relationship between plant diversity and productivity: resource complementarity among plants and plant–soil feedbacks (Barry et al., 2019). Negative plant–soil feedbacks are known to be high at low diversity causing loss of productivity, while positive plant–soil feedbacks may dominate at high diversity (Eisenhauer et al., 2012; Kulmatiski et al., 2008; van Ruijven et al., 2020). Next to these two main drivers, there are many more factors that may influence the relationship between diversity and productivity. Important negative influences are aboveground pests, such as leaf pathogens (Clarke & Eagling, 1994) and herbivores (Crawley, 1989), which were shown to change in abundance and diversity with decreasing plant species richness (Ebeling et al., 2014; Mitchell et al., 2002; Rottstock et al., 2014). Moreover, changes in abiotic conditions along the plant species richness gradient can influence plant community productivity (Wright et al., 2017). Previous studies demonstrated, for example, changes in the availability of nutrients and light with decreasing plant species richness (Bachmann et al., 2018; Lange et al., 2019; Oelmann et al., 2011). As a result, these changes in environmental conditions may influence phenotypic trait expression, reproductive success, and fitness of plants (Gubsch et al., 2011; Scherber et al., 2006; Schmidtke et al., 2010).

Taken together, multiple interactions between organisms and plant phenotypic trait expression and fitness are changing along the plant diversity gradient. This raises the question about eco‐evolutionary feedbacks, so that populations of the same plant species would differently develop over time when growing at high or low diversity (Bailey et al., 2006; Linhart, 1988; Post & Palkovacs, 2009). In fact, it was shown that soil organisms influence selection patterns of plants (Lau & Lennon, 2011), so that changes in plant–soil feedbacks could alter the selection environment and thus lead to shifts in the micro‐evolution of the plants (Zuppinger‐Dingley et al., 2016). Moreover, it was shown that plants in high‐diversity communities were selected for greater niche complementarity among species (Zuppinger‐Dingley et al., 2014), promoting the coexistence of multiple species over time. In contrast, the accumulation of soil‐borne pathogens in low‐diversity communities may change the selection environment to favor plant individuals that are able to reduce negative plant–soil feedbacks, for example, through increased pathogen resistance (Karasov et al., 2017).

To date, changes in plant–soil feedbacks and eco‐evolutionary pathways are assumed to be the main drivers of change in the productivity–diversity relationship (Eisenhauer et al., 2019); however, their joint influence is poorly studied under field conditions (Vogel et al., 2019). We are only aware of two studies addressing this issue by using a reciprocal transplantation approach in biodiversity experiments (Kleynhans et al., 2016; Lipowsky et al., 2011). Both studies provided first empirical evidence for divergence of plants into low‐ and high‐diversity ecotypes. However, model plants were transplanted into intact plant communities, which makes it hard to differentiate between the effects of soil biota and conditions on the one hand and influences of plants growing in the vicinity on the other hand. Moreover, the transplantation was done either after a relative short period of selection (after 5 years) (Lipowsky et al., 2011) or only a single plant species was used for the experiment (Kleynhans et al., 2016).

Here, we tested how low and high plant diversity and associated differences in soil biota and conditions influence eco‐evolutionary feedbacks of plants by performing a reciprocal transplant experiment in a long‐running grassland biodiversity experiment (Jena Experiment) (Roscher et al., 2004; Weisser et al., 2017). We used two‐ and six‐species communities, where plants had been growing over a period of 14 years, and collected seeds from four grass species. Before we planted the offspring as “phytometers”, we removed the vegetation of the study plots to exclude plant–plant interactions and to deliberately study the response of the phytometers to soils with a different history. In a reciprocal design, we transplanted five “home” phytometers (same origin and actual environment); five “away‐same” phytometers (same species richness of origin and actual environment, but different plant composition), and five “away‐different” phytometers (different species richness of origin and actual environment) of the same plant species in each of the research plots. We measured performance (survival, biomass production), fitness (number of inflorescences), leaf traits related to relative growth rates (leaf greenness, specific leaf area), and leaf damage by pathogens and herbivores. We hypothesized that

1. plants developed differently over time due to different plant–soil feedback effects at low and high diversity. Therefore, we expected that plants in home soil have a higher biomass production and fitness than those in away soil.
2. plants growing in low‐diversity communities are selected for lower productivity and fitness due to higher investment into enemy defense. Thus, offspring of plants from low‐diversity communities should have lower biomass production, fitness, and leaf damage than offspring from high‐diversity communities, regardless of the actual environment. Further, we expected that plants from high‐diversity plots show higher leaf greenness and specific leaf area as traits indicating higher relative growth rates.
3. low‐diversity communities had a higher accumulation of soil‐borne pathogens than high‐diversity communities causing stronger negative plant–soil feedback effects. We expected that plants transplanted in low‐diversity plots show lower productivity and fitness than those transplanted in high‐diversity plots, regardless of their origin environment.

​

## 2 MATERIALS AND METHODS

### 2.1 Study site—Jena Experiment

The Jena Experiment is a long‐term biodiversity experiment located in the floodplain of the Saale River near Jena (Thuringia, Germany, 50°55′N, 11°35′E, 130 m a.s.l.) (Roscher et al., 2004). The area around Jena has a mean annual air temperature of 9.9°C and an annual precipitation of 660 mm (1980–2010) (Hoffmann et al., 2014). During the first experimental year of this study (2018), Central Europe experienced a severe summer drought. At the experiment site, the annual precipitation was 32.9% (395.3 mm) lower than the average for 2007–2017 (589.3 mm), and the mean annual air temperature was 0.9°C (10.6°C) higher than for 2007–2017 (9.7°C; weather station Jena‐Saale, Max Planck Institute for Biogeochemistry Jena, https://www.bgc‐jena.mpg.de/wetter/; Figure S1). Precipitation was particularly low in the summer months (69.9% less than the 2007–2017 average for June, July, and August; Figure S1), while mean air temperature in summer (June, July, August) generally increased from 2007 to 2019 (simple linear regression: *R* ^2^ = 0.59, *p* = 0.002; Figure S1). In summer 2019, the precipitation was 34% lower than the average for 2007–2017, and the summer mean air temperature further increased compared with 2018 (Figure S1).

The Jena Experiment was established in 2002 on a former highly fertilized arable field, which had been used for growing wheat and vegetables from the early 1960s until 2000. The soil of the study site is a Eutric Fluvisol. Soil texture changes from sandy loam to silty clay with increasing distance from the river. Thus, four blocks were arranged parallel to the riverside (Roscher et al., 2004). The present study was carried out in a subexperiment of the Jena Experiment, the so‐called dominance experiment. This experiment included nine plant species, which often reach dominance in Central European mesophilic grasslands of the Arrhenatherion type (Ellenberg, 1988): five grasses, two legumes, and two herbs. This design was chosen, because it may represent conditions where only/mostly dominant species prevail, such as in many fertilized grasslands in Central Europe. Species were grown in plots of 3.5 × 3.5 m from 2002 to 2009 until plot size was reduced to 1 × 1 m (2010–2018). Species richness levels ranged from one to nine species (1, 2, 3, 4, 6, and 9 plant species; for more information, see Roscher et al., 2004). Seeds were sown with a density of 1,000 viable seeds per m^2^ in early May 2002. Seeds from all species were purchased from a commercial supplier (Rieger‐Hofmann GmBH), who collected seeds from natural habitats, which were grown and propagated for a maximum of 5 years providing a genetic diversity similar to those expected from natural grasslands. As it is typical for traditional management of extensive hay meadows in the study region, plots were mown every year in June and September and mown plant material was removed. Plots were regularly weeded to maintain the experimental species combinations from 2002 to 2017. No fertilizer was added during the experiment.

### 2.2 Selection of study species and research plots

In summer 2016, all available ripe seeds of the nine species were collected in all plots and stored as bulk samples (per species and plot) in a freezer (−20°C) to maintain their viability until the start of the experiment. Because several species went extinct over the years or showed an insufficient seed production in 2016, four grass species (*Alopecurus pratensis* L., *Arrhenatherum elatius* (L.) P. Beauv. ex J. Presl et C. Presl, *Dactylis glomerata* L., and *Poa trivialis* L.) were chosen for this study. For every species, six two‐ and six six‐species communities were selected, where the respective species produced a sufficient amount of seeds in 2016 (*N* = 48 plots). These plant diversity levels represent contrasted biotic conditions that plant species may experience in their vicinity in fertile grasslands. The chosen plots were evenly distributed in the four blocks.

The four grasses (family *Poaceae*; subfamily *Pooideae*) are perennial and wind‐pollinated species, but differ in growth forms (Mühlberg, 1967; Roscher et al., 2004). *Arrhenatherum elatius* forms tussocks and is able to form sterile leafy shoots (Mühlberg, 1967; Pfitzenmeyer, 1962). *Dactylis glomerata* also grows as tussock (Mühlberg, 1967; Roscher et al., 2004). *Alopecurus pratensis* grows with belowground stolons, while *P. trivialis* grows with aboveground creeping shoots (Mühlberg, 1967). Furthermore, *P. trivialis* is characterized by a low growth height and a shallow rooting system compared with the other grass species (Haggar, 1971). Among the study species, *A. elatius* and *D. glomerata* were the most productive species in the mixtures of the dominance experiment over the last 14 years, while *A. pratensis* and *P. trivialis* showed intermediate levels and decreased in productivity over the years (Figure S2) (Clark et al., 2019; Roscher et al., 2007).

### 2.3 Preparation of phytometer plants

QuickPot™ trays of 20 cm^3^ volume (Hermann Meyer KG) were sterilized with a potassium hypochlorite solution (Eau de Javel: 2.6 g KClO to 100 ml water; 1:1) and filled with a sterilized sand–soil mix (1:1; soil originated from the field site; autoclaved twice for 40 min at 121°C). Each species and origin (i.e., plot) were sown with two or three seeds per pot, starting with *A. pratensis* (22 January 2018), followed by *D. glomerata* (23 January 2018), *P. trivialis* (26 January 2018), and *A. elatius* (29 January 2018). QuickPot™ trays were placed in a greenhouse (16‐hr day at 20°C and 8‐hr night at 12°C) and regularly watered (with demineralized water). On 5 February, *A. pratensis* seeds were reseeded, because of a low germination rate. For the other three species, one or two seedlings per pot were removed if more than one seed had germinated. After some weeks of growth (12 March and 5 April 2018), seedlings were cut 6 cm above ground to promote tillering. Before the first cut, height and shoot number of seedlings originated from two‐ and six‐species communities were not significantly different (data not shown).

### 2.4 Establishment of the phytometer experiment

**FIGURE 1:**
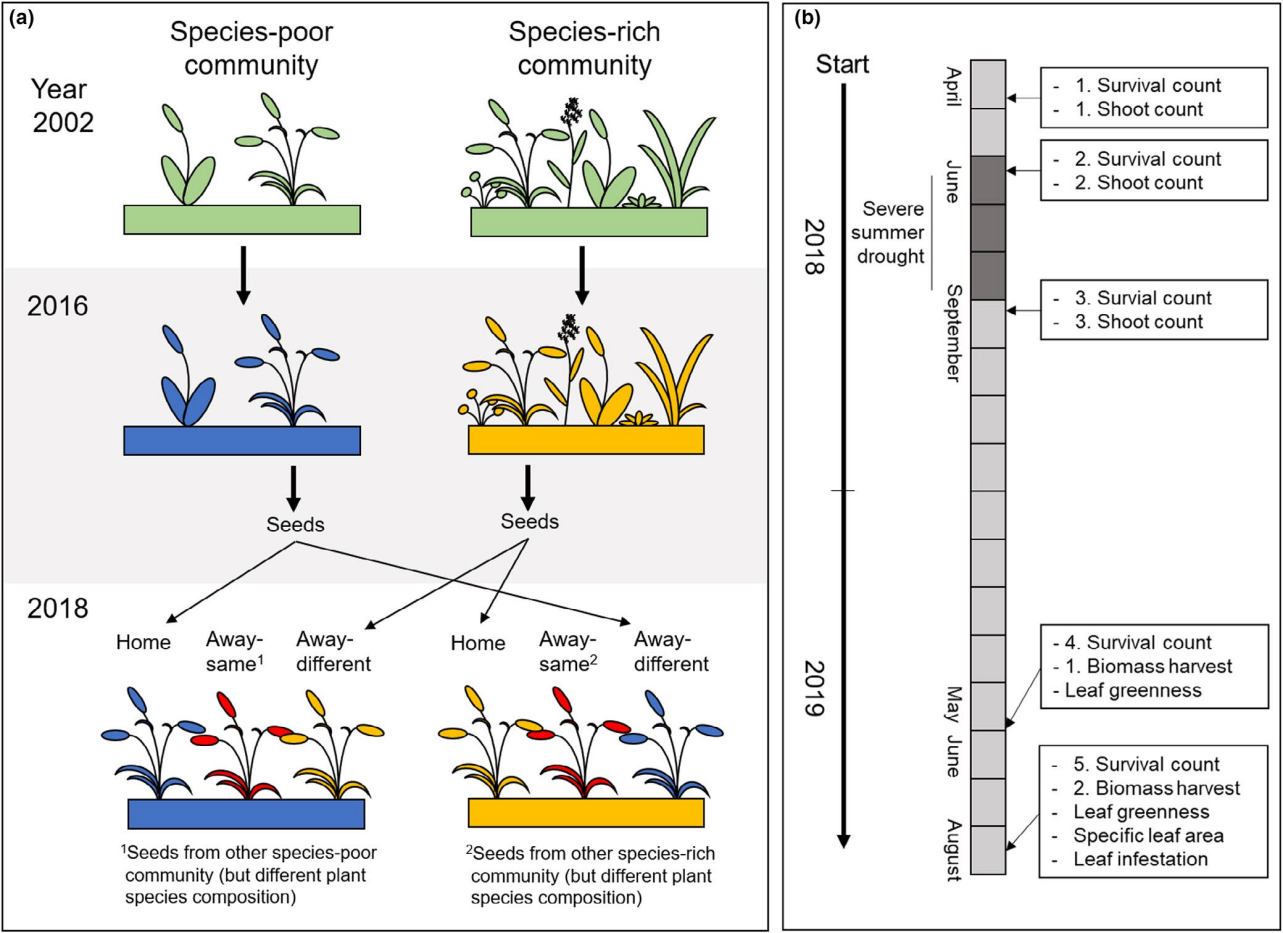
Overview of experimental design (a) and chronological sequence of the data collection (b). In 2016, ripe seeds were collected from two‐species communities (blue‐colored communities; a) and six‐species communities (yellow‐colored communities; a). Starting in April 2018, the plant sod of the plots was removed and 15 seedlings, germinated from collected seeds, were transplanted in a 20 × 20 cm grid in each plot. In a reciprocal design, we transplanted five “home” phytometers (same origin and actual environment, indicated by similar color of plant and soil), five “away‐same” phytometers (same species richness of origin and actual environment, but different plant composition; indicated by red‐colored plants), and five “away‐different” phytometers (different species richness of origin and actual environment, indicated by different color of plant and soil) of the same species in the study plots. The phytometers were grown for two years

On 9 April 2018, the plant sod was removed with a digger on the 48 selected plots of the dominance experiment. To remove larger roots, the soil was mixed and homogenized to 30 cm depth with the digger (such as also done in Vogel et al., 2019). Afterward, 15 seedlings of the target species were transplanted in a 20 × 20 cm grid in each plot. Five seedlings originated from the same plot (“home” soil), which means that seeds were obtained from plants growing on this plot, while the other 10 seedlings originated from other plots (“away” soil). Five of these “away” seedlings originated from seeds obtained from five randomly chosen two‐species communities, and five of these seedlings originated from five randomly chosen six‐species communities containing these species (Figure 1a). Depending on the species richness of plot‐specific community, “away” plants were classified as “away‐same” (species richness of the origin community and species richness of actual community were the same, but plant composition was different) or as “away‐different” (species richness of origin community and species richness of actual community were different). The positions of the “home,” “away‐same,” and “away‐different” seedlings within the grid were determined by chance. Starting on 16 April 2018, seedlings of *D. glomerata* were transplanted, followed by *A. pratensis* (17 April 2018), *A. elatius* (17 and 18 April 2018), and *P. trivialis* (18 April 2018). In total, 720 phytometer individuals were planted. Plants were watered directly after the transplantation and then every other day until the end of April. Plots were weeded every month from May to October 2018 and three times from April to July 2019. To mimic mowing, phytometers were cut 15 cm above ground on 27 September 2018.

### 2.5 Data collection

#### 2.5.1 Survival and aboveground biomass production

We measured survival and biomass production of plants at regular intervals over the two years to determine the potential effects of the extreme summer drought in 2018 and to account for potential changes in plant–soil feedbacks with plant aging. Survival was recorded five times—in April (24–27 April), June (01–08 June) and September (04–05 September) 2018, and May (22–23 May) and August (27–29 August) 2019 (for chronological sequence, see Figure 1b). A phytometer was defined as alive when the plant had at least one living shoot with green leaves. In order to avoid underestimation of survival, we classified “dead” phytometers that had resprouted in the next growing season as alive. Living shoots, as proxy for aboveground biomass production (Roscher, Schumacher, Weisser, et al., 2007), were counted in April, June, and September 2018 (same days as for survival counting; Figure 1b). Due to the extreme summer drought in 2018, we did not harvest aboveground biomass in August 2018, contrary to our initial planning, in order to prevent further damage to the plants. In 2019, phytometer plants were harvested in spring, starting with *D. glomerata* (28 May), followed by *A. pratensis* (29 May), *A. elatius* (3 June), and *P. trivialis* (4 June); and in summer, starting with *A. elatius* (27 August), followed by *D. glomerata* (28 August), and *A. pratensis* (29 August; *P. trivialis* had become extinct in August 2019; Figure 1b). Plants were cut three centimeters above ground level, were dried at 70°C for 48 hr, and weighed.

#### 2.5.2 Number of inflorescences and leaf traits of phytometers

Inflorescences per phytometer were counted in May 2019, because the grass species mainly flower in early summer. Furthermore, leaf greenness (=unitless estimate of foliar chlorophyll content) of three leaves from different shoots per individuals was measured with a SPAD 502 Plus Chlorophyll Meter (Spectrum Technologies, Inc.). For *A. elatius*, leaves (i.e., third leaf from above) of reproductive shoots were measured, while leaves of vegetative shoots were measured for *A. pratensis* and *D. glomerata*. The small and short leaves of *P. trivialis* individuals in 2019 made in situ measurements in the field impossible. Therefore, three leaves from reproductive shoots per individual (if possible) were collected on 4 June 2019, which were covered in wet tissues and stored in a fridge (4°C) over night. On 5 June 2019, water droplets were removed with tissue and leaf greenness was measured in the laboratory.

Before the second harvest (starting on 12 August 2019), five leaves from different vegetative shoots per individual (except *P. trivialis*) were collected, which were stored in a cooling box for transport. In laboratory, leaf greenness was measured with SPAD 502 Plus Chlorophyll Meter (Spectrum Technologies, Inc.) and total leaf area (mm^2^_leaf_) was measured with a leaf area meter (LI‐3100 Area Meter, LI‐COR, USA). Leaf samples were dried for 48 hr at 70°C and weighed, and specific leaf area (SLA; mm^2^_leaf_ mg^−1^_dw_) was calculated as ratio between total leaf area and total leaf mass. Plant weight and leaf weight per individual were summed up to get total biomass for the August harvest 2019.

#### 2.5.3 Leaf infestation and herbivory

Leaf samples collected in August 2019 were further used to record leaf damages (before drying). Due to the specificity of the symptoms, physiological disorders and viral and bacterial infections could be excluded as the main cause for the leaf damages observed. Instead, fungi and arthropods were identified as the causative agents of leaf area loss and of chlorosis and necrosis. The area, which was damaged by fungi or arthropods, respectively, was estimated by comparing the damaged leaf area to circular and square templates ranging in size from 1 to 500 mm^2^ (Meyer et al., 2017). For *A. pratensis* and *D. glomerata*, additionally dead leaf area was estimated (leaf tip senescence) and subtracted from total leaf area. The ratio between total leaf area and damaged leaf area was calculated as measure of fungal infestation (%) and herbivory (%), respectively. The majority of fungal pathogens cannot be directly determined by eye, but individual pathogen groups can be comparatively easily assigned due to their distinct morphological characteristics partly in connection with typical necrotic changes in the leaf tissue. Leaf fungi of a subsample of leaves per species were identified morphologically to the species level using a light microscope.

### 2.6 Statistical analysis

In order to test whether phytometer plants differ in survival depending on plant and soil history, we used generalized linear mixed‐effects models with a logit link function, and survival (dead or alive) modeled as a binary response variable. However, due to the high survival rates of *A. pratensis, A. elatius*, and *D. glomerata* over the duration of the entire experiment and no survival at all of *P. trivialis* during the fifth count (August 2019), we only analyzed the survival of *P. trivialis* during the counts three (September 2018; 75% survival) and four (May 2019; 55% survival). Block and plot identity (i.e., plot, where the phytometers were planted) nested in block were used as random effects in the model. We started with a null model with the random effects only and added the fixed effects in the following order: species richness of the origin community (origin species richness (origin SR)), species richness of the actual plot (i.e., where the phytometers were planted; actual species richness (actual SR)), soil treatment (three levels: home, away‐same, and away‐different), season (September 2018, May 2019), and the interactions between variables and season (origin SR × season; actual SR × season; soil treatment × season).

To test whether the phytometer plants performed differently depending on plant and soil history, linear mixed‐effects models were fitted with number of shoots, biomass production, number of inflorescences, leaf traits (greenness, SLA), and leaf fungal infestation as response variables, respectively. Herbivory was not analyzed, because only 1% of the measured leaves were affected (27 out of 2,539 leaves). Prior to statistical analyses, response variables were averaged per plot and soil treatment (home, away‐same, away‐different) to eliminate the expected higher variance in the away treatments (each away plant per plot came from a different origin plot). Variables were transformed to meet the assumptions of normality and variance homogeneity: Shoot number and leaf fungal infestation were log‐transformed; biomass production, SLA, and number of inflorescences were square‐root‐transformed. We used again block and actual plot identity nested in block as random effects and started with a null model with the random effects only. Then, we successively added the fixed effects with species identity first, followed by the treatment variables (origin SR, actual SR, soil treatment). In case of shoot number, biomass production and leaf greenness, season, and all possible combinations of species ID, treatment variables, and season were additionally entered. To test whether the initial size influenced the performance of the phytometers later in the experiment, we added shoot number in April 2018 at planting as a covariate before the other fixed effects in a separate model (for analysis of shoot number). All models were performed with four and with three species (without *P. trivialis*) to check for possible bias caused by the high mortality of *P. trivialis* (except for response variables measured in June 2018 and August 2019). We further analyzed the response variables separately per season, because there was either a significant interaction among treatment variables and season (number of shoots) or only three species were included in August 2019 due to the extinction of *P. trivialis* (biomass production, leaf greenness). Moreover, if the interaction among species ID and treatment variables was significant in these models, we further analyzed the data for each species separately. We used the same model structure as described above, but without season and species ID (for analysis at species level) as fixed effects. To test for the relationship between biomass production and number of inflorescences in May 2019, we used species identity, biomass production, and their interaction as fixed effects in a separate model with number of inflorescences as response variable (and again block and plot identity nested in block as random effects). All models were fitted with maximum likelihood (ML), and likelihood ratio tests were used to decide on the significance of the fixed effects. Differences among treatment groups were tested with Tukey's HSD test. All calculations and statistical analyses were done in R (version 3.6.1, R Development Core Team, http://www.R‐project.org) including the package *lme4* (glmer and lmer) (Bates et al., 2015) and *multcomp* (Tukey HSD) (Hothorn et al., 2008) for mixed‐effects model analysis.

## 3 RESULTS

### 3.1 Survival of phytometers

Survival rates of *A. pratensis, A. elatius*, and *D. glomerata* phytometers were nearly 100% (>96%) after one year of growth (April 2018–May 2019). The final record in August 2019 indicated a slightly increased mortality of *A. pratensis* (92% survival), while survival of *A. elatius* and *D. glomerata* phytometers was still high (98%). All phytometers of *P. trivialis* survived until June 2018, but mortality increased in summer 2018 (25% mortality in September 2018). In May 2019, we recorded 45% mortality, while in August 2019 all *P. trivialis* phytometers were dead.

**FIGURE 2:**
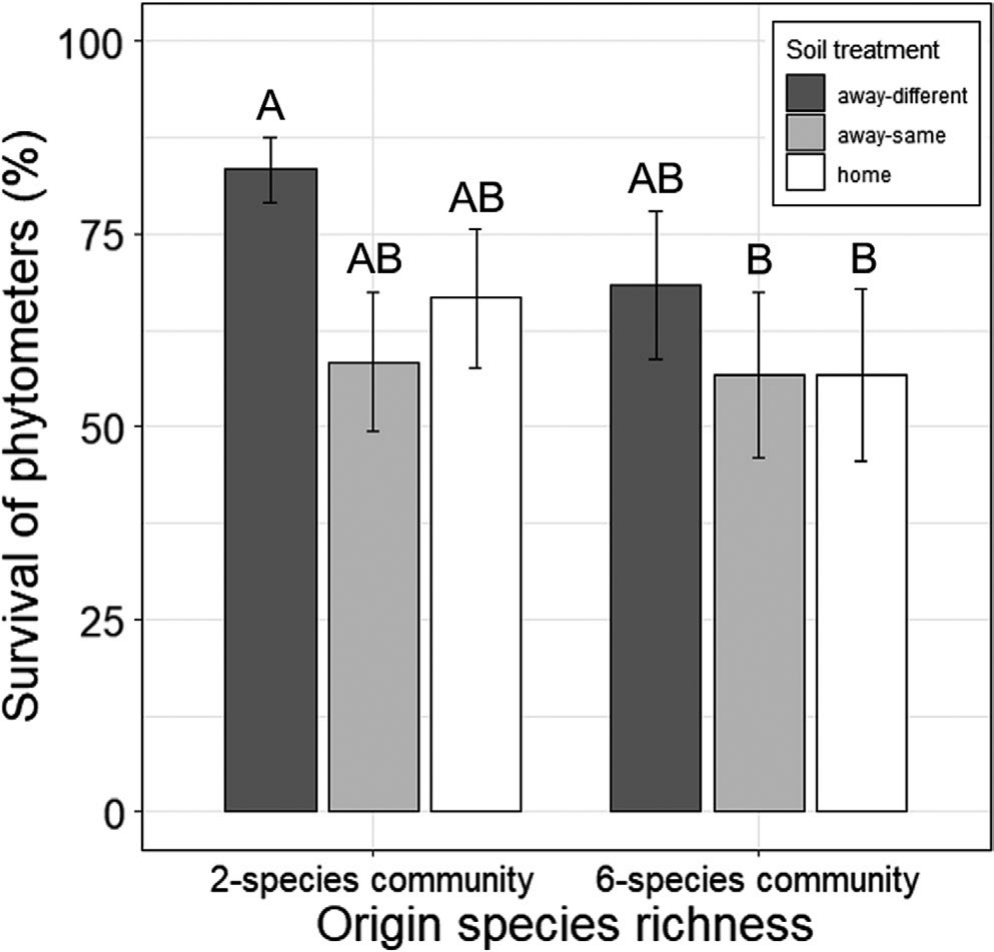
Survival of *Poa trivialis* phytometers originated from two‐ and six‐species communities (origin species richness) transplanted in away‐different, away‐same, and home soil (soil treatment; averaged for September 2018 and May 2019). Bars show mean values (±1 SE); letters above bars indicate significant (*p* < 0.05) differences among categories (Tukey's HSD test)

*Poa trivialis* phytometers originating from two‐species communities had a higher survival than plants from six‐species communities (until May 2019; Table 1). Moreover, phytometers in away‐different soil showed higher survival than plants in home soil and away‐same soil (Table 1). These results were mainly due to a high survival of offspring originating from two‐species communities transplanted into soil of six‐species communities (Figure 2). Other groups (away‐different with origin in 6‐species communities, away‐same, and home) had an equally lower survival (Figure 2).

**TABLE 1:** Summary of generalized linear mixed‐effects model analyses testing the effects of origin species richness (origin Sr; two‐ or six‐species community), actual species richness (actual Sr; two‐ or six‐species community), soil treatment (away‐different, away‐same, home), season (September 2018, May 2019), and their interactions on the survival of *Poa trivialis* phytometers

	*Df*	Chi^2^	*p*
Origin Sr	4	4.73	**0.030**
Actual Sr	5	0.14	0.705
Soil treatment	7	14.45	**0.001**
Season	8	21.81	**<0.001**
Origin Sr × season	9	0.03	0.871
Actual Sr × season	10	3.49	*0.062*
Soil treatment × season	12	0.49	0.783

Note

Shown are degrees of freedom (*Df*), Chi^2^, and *p*‐values (*p*). Significant factors and interactions are given in bold, and marginally significant effects are given in italics.

### 3.2 Shoot number and biomass production of phytometers

**FIGURE 3:**
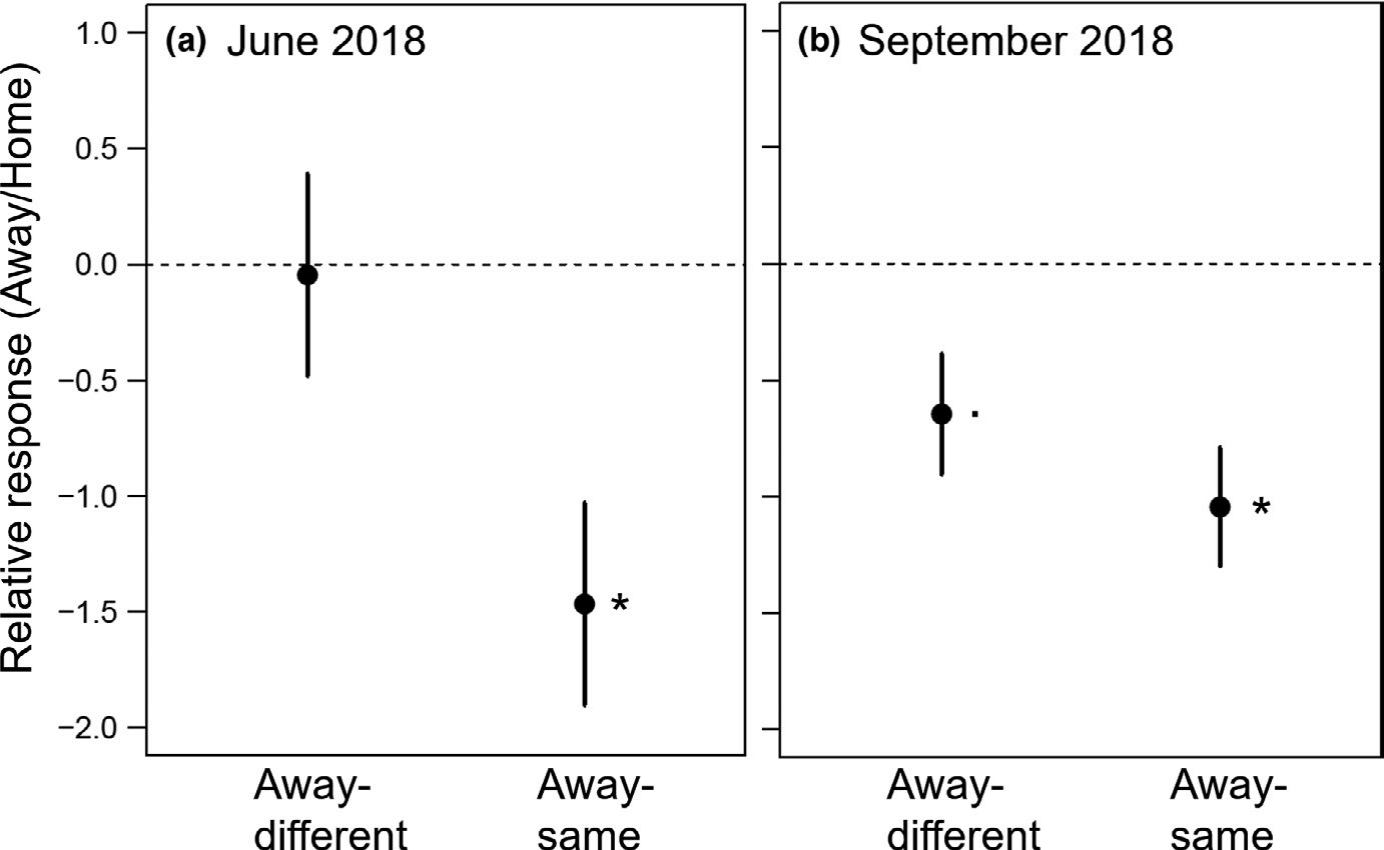
Shoot production of phytometers transplanted in away‐different and away‐same soil relative to phytometers in home soil (soil treatment) in (a) June and (b) September 2018. Points are means (over all four species; ±1 SE). Negative values indicate a lower shoot production of plants in away soil compared with plants in home soil, and positive values indicate a higher shoot production. Stars show significant differences between home and away plants, and dots show marginally significant differences

The initial number of shoots had no influence on the further performance in 2018 (Table S1). Number of shoots varied depends on soil treatment in spring and summer 2018, respectively (Table 2). In June, home and away‐different phytometers produced a higher number of shoots than away‐same phytometers (Figure 3a). The shoot number of *A. elatius* in soil of two‐species communities was higher compared with those in six‐species communities (significant interaction species ID × actual species richness), while shoot number of other species did not differ depending on actual species richness (Table 2; Table S2). In September, home phytometers had a higher number of shoots than away‐same and away‐different plants (Figure 3b). Moreover, *D. glomerata* had higher number of shoots when originated from 6‐species communities, while *P. trivialis* produced more shoots when originated from 2‐species communities (Table S2), causing the significant interaction of species ID × origin species richness in September 2018 (Table 2).

**TABLE 2:** Summary of linear mixed‐effects model analyses testing the effects of species identity (species ID), origin species richness (origin Sr; two‐ or six‐species community), actual species richness (actual Sr; two‐ or six‐species community), soil treatment (away‐different, away‐same, home), and their interactions on number of shoots in June and September 2018 (in September with and without *Poa trivialis*, because of high mortality)

	June 2018			September 2018					
	All species			All species			Without *P. trivialis*		
	*Df*	Chi^2^	*p*	*Df*	Chi^2^	*p*	*Df*	Chi^2^	*p*
Species ID	7	143.49	**<0.001**	7	44.74	**<0.001**	6	5.14	**<0.001**
Origin Sr	8	3.14	*0.076*	8	0.76	0.382	7	0.21	0.651
Actual Sr	9	0.46	0.497	9	0.20	0.655	8	0.05	0.816
Soil treatment	11	6.75	**0.034**	11	7.67	**0.022**	10	10.74	**0.005**
Species ID × origin Sr	14	0.94	0.816	14	14.89	**0.002**	12	8.44	**0.015**
Species ID × actual Sr	17	9.28	**0.026**	17	6.58	*0.086*	14	4.87	*0.088*
Species ID × soil treatment	23	9.77	0.135	23	7.26	0.297	18	6.80	0.147

Note

Shown are degrees of freedom (*Df*), Chi^2^, and *p*‐values (*p*). Significant factors and interactions are given in bold, and marginally significant effects are given in italics.

**FIGURE 4:**
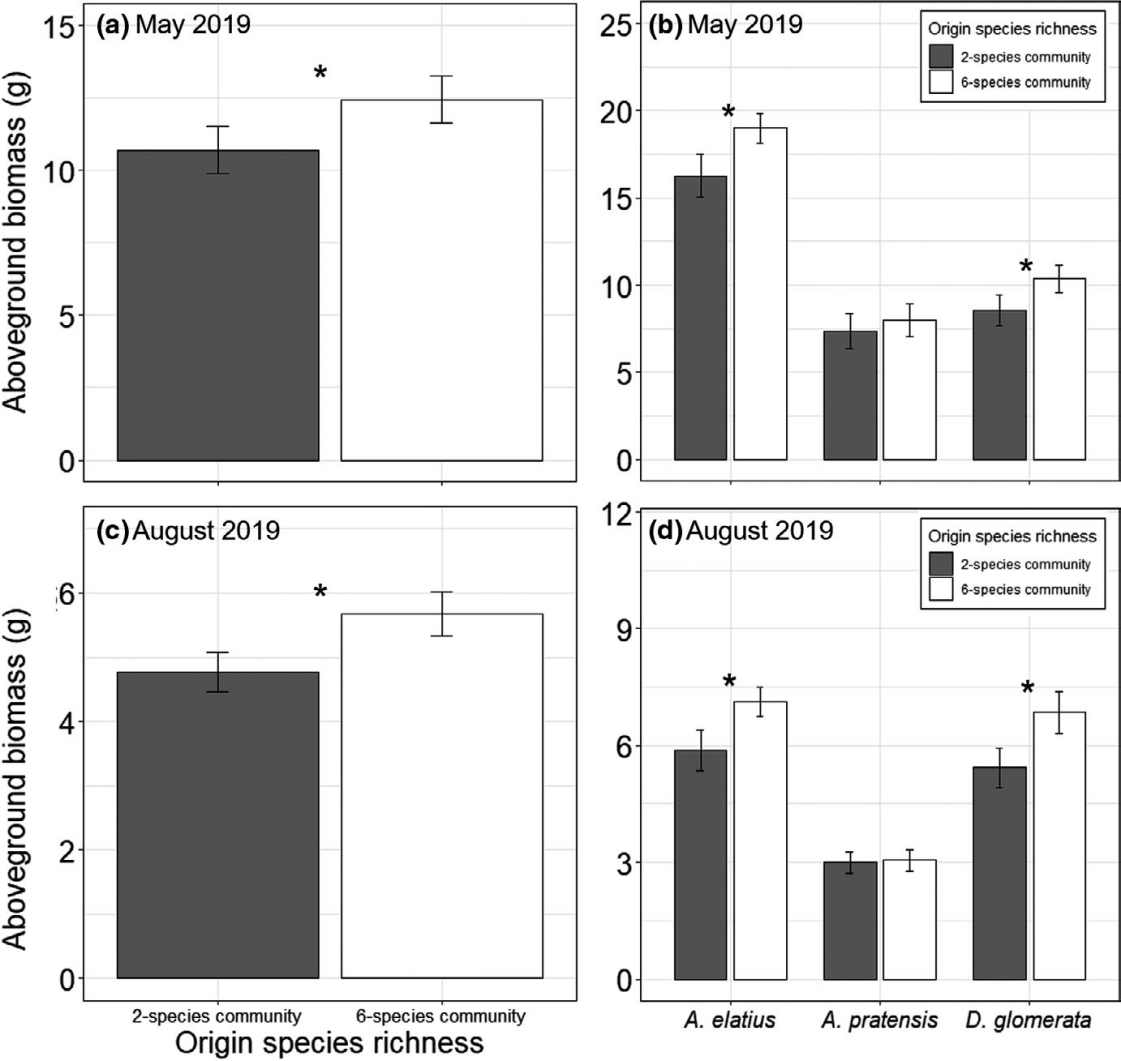
Aboveground biomass production of phytometers originated from two‐ and six‐species communities (origin species richness) across all species (a) and separately for each species (b) in May 2019, and across all species (c) and separately for each species (b) in August 2019. Bars show mean values (±1 SE); stars above bars indicate significant (*p* < 0.05) differences

**FIGURE 5:**
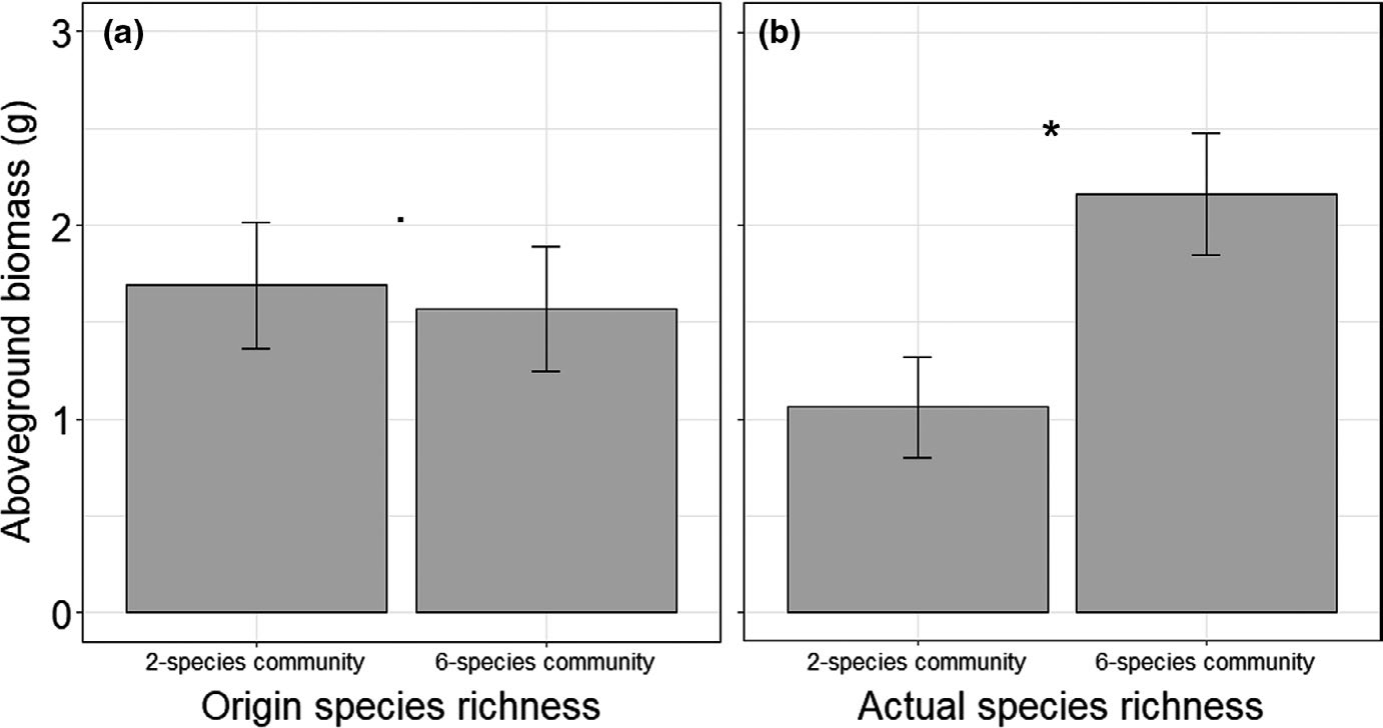
Aboveground biomass production of *Poa trivialis* phytometers, when originated from two‐ and six‐species communities (origin species richness; a) and transplanted in soil of two‐ and six‐species communities (actual species richness; b) in May 2019. Bars show mean values (±1 SE); a star above bars indicates significant (*p* < 0.05) differences, and a dot indicates marginal significant differences (*p* < 0.065)

In the second year of the experiment, aboveground biomass production varied significantly dependent on origin species richness in spring and summer (Table 3). In both seasons, phytometers originating from six‐species communities generally produced more biomass than phytometers from two‐species communities (Figure 4). One exception is *P. trivialis* in May 2019, which tended to produce more biomass when originated from two‐species communities, causing the significant interaction of species ID × origin species richness in May 2019 (Table 3; Figure 5a; Table S3). Furthermore, in May, *P. trivialis* produced more biomass when transplanted in soil of six‐species communities than in soil of two‐species communities (Figure 5b; Table S3).

**TABLE 3:** Summary of linear mixed‐effects model analyses testing the effects of species identity (species ID), origin species richness (origin Sr; two‐ or six‐species community), actual species richness (actual Sr; two‐ or six‐species community), soil treatment (away‐different, away‐same, home), and their interactions on aboveground biomass production in May and August 2019 (in May with and without *Poa trivialis*, because of 45% mortality; in August without *P. trivialis*, because of 100% mortality)

	May 2019						August 2019		
	All species			Without *P. trivialis*			Without *P. trivialis*		
	*Df*	Chi^2^	*p*	*Df*	Chi^2^	*p*	*Df*	Chi^2^	*p*
Species ID	7	83.59	**<0.001**	6	35.51	**<0.001**	6	42.88	**<0.001**
Origin Sr	8	4.05	**0.044**	7	7.75	**0.005**	7	7.79	**0.005**
Actual Sr	9	1.78	0.182	8	0.26	0.613	8	0.15	0.701
Soil treatment	11	4.07	0.130	10	4.19	0.123	10	2.62	0.269
Species ID × origin Sr	14	9.59	**0.022**	12	2.68	0.262	12	4.02	0.134
Species ID × actual Sr	17	4.15	0.246	14	2.42	0.299	14	1.63	0.443
Species ID × soil treatment	23	5.24	0.513	18	4.15	0.387	18	4.06	0.398

Note

Shown are degrees of freedom (Df), Chi^2^, and p‐values (P). Significant factors and interactions are given in bold, and marginally significant effects are given in italics.

### 3.3 Number of inflorescences and leaf traits of phytometers

Number of inflorescences was higher when plants originated from six‐species communities (12.87 ± 1.11 (SE)) than from two‐species communities (11.48 ± 1.11) when *P. trivialis* was excluded from the analysis (marginal significant; Table 4). Leaf greenness and specific leaf area did not differ depending on plant or soil history (Tables S4; S5). The significant interaction of species ID × soil treatment on leaf greenness in spring 2019 (with four species) was caused by a higher leaf greenness of *P. trivialis* in home soil than in away‐same soil, while other species showed no difference among the soil treatments (Tables S4; S6; Figure S3). Biomass production in May 2019 was positively correlated with number of inflorescences (Table S7; Figure S4).

**TABLE 4:** Summary of linear mixed‐effects model analyses testing the effects of species identity (species ID), origin species richness (origin Sr; two‐ or six‐species community), actual species richness (actual Sr; two‐ or six‐species community), soil treatment (away‐different, away‐same, home), and their interactions on number of inflorescences in May 2019. Models were run for four and three species (with and without *Poa trivialis*, because of 45% mortality)

	All species			Without *P. trivialis*		
	*Df*	Chi^2^	*p*	*Df*	Chi^2^	*p*
Species ID	7	33.57	**<0.001**	6	31.97	**<0.001**
Origin Sr	8	0.41	0.523	7	3.57	*0.059*
Actual Sr	9	2.52	0.112	8	0.48	0.487
Soil treatment	11	2.72	0.257	10	2.16	0.340
Species ID × origin Sr	14	5.74	0.125	12	1.45	0.484
Species ID × actual Sr	17	4.83	0.185	14	3.07	0.216
Species ID × soil treatment	23	4.32	0.634	18	3.97	0.409

Note

Shown are degrees of freedom (*Df*), Chi^2^, and *p*‐values (*p*). Significant factors and interactions are given in bold, and marginally significant effects are given in italics.

### 3.4 Leaf fungal infestation and herbivory

Almost all leaves of phytometers showed damage by fungi (97%), while only 1% of measured leaves (*N* = 2,539) were affected by herbivory. In *A. elatius* and *D. glomerata,* the pathogen *Puccinia coronata* Corda was identified as main cause of fungal damage, which was partly hyperparasitized by *Eudarluca caricis* (Fr). O.E. Erikss. in *D. glomerata*. In *A. pratensis*, leaves often showed stronger necrosis that were presumably also caused by *P. coronata*. Plant or soil history had no significant influence on leaf infestation (Table S5).

## 4 DISCUSSION

In the present study, we tested how low and high plant diversity and associated changes in plant–soil feedbacks over time influence eco‐evolutionary feedbacks of plants. Therefore, we performed a reciprocal transplant experiment in plant communities of a long‐term biodiversity experiment (Jena Experiment). We hypothesized that the performance of phytometers is higher when transplanted in soil of the home community than in soil of a different community (Hypothesis 1). Moreover, we assumed that offspring of plants from high‐diversity communities produce more biomass (Hypothesis 2) and that plant biomass production is generally higher when transplanted in soil of high‐diversity communities (Hypothesis 3). In the establishment year, our results supported Hypothesis 1 by showing that plants in home soil had a higher performance (higher number of shoots) than in away soil. In the second year, the “home advantage” disappeared, but offspring of plants selected at higher diversity produced more biomass than the offspring of plants selected at low plant diversity, supporting Hypothesis 2. In both years, we found little evidence for Hypothesis 3. Our results provide first empirical evidence that eco‐evolutionary feedbacks change with plant diversity. Our findings could be critical to better understand the strengthening positive diversity–productivity relationships found in long‐term biodiversity experiments.

Hypothesis 1 Phytometers perform better in home than in away soil.

In the establishment year, phytometer plants in home soil were more productive than phytometers in away soil. This home advantage supports the idea that a change in plant community diversity and associated changes in plant–soil feedback effects alter the selection environment for plants. Consequently, eco‐evolutionary feedbacks differ at low and high diversity, which is in line with Lipowsky et al. (2011) and Kleynhans et al. (2016), and probably cause low‐diversity and high‐diversity genotypes as shown in van Moorsel et al. (2019). In addition to this, the lower performance of plants in away‐same than in home soil highlights that both plant species diversity and plant species composition determine plant–soil feedback effects and thus the selection environment (De Long et al., 2020; He & Lamont, 2010). Nevertheless, we can only speculate about the exact reasons for the home advantage. Probably, home plants better interact with their associated soil community, as it was shown in previous greenhouse studies (Johnson et al., 2010; Pregitzer et al., 2010; Wagg et al., 2015). Additionally, it is also possible that plants are better able to cope with given soil properties at high or low diversity, as shown, for example, by the different availability of specific nitrogen and phosphorus compounds along the species richness gradient (Hacker et al., 2015; Vogel et al., 2019). In the second year of the experiment, the home advantage disappeared, which means that soil biota had a lower direct influence on the performance of adult plants. This is in line with a study by Dudenhoffer et al. (2018) showing that significant plant–soil feedback effects at juvenile stages of plants became neutral at later growth stages.

Hypothesis 2 Phytometers originated from high‐diversity communities show higher performance, but also higher leaf damage, than phytometers from low‐diversity communities.

At later growth stages (in 2019), the performance of the phytometers significantly differed depending on plant history: Offspring of plants selected at high diversity had a higher biomass production and fitness than offspring of plants selected at low diversity, regardless of the soil in which they were planted. This demonstrates that the selection environment at high diversity may lead to more stable populations (higher fitness) than at low diversity. Thus, our results provide one of the few empirical evidences to date that the strength in the diversity–productivity relationship is influenced by different selection at low and high diversity causing measurable differences in the plant phenotype.

At the level of individual species, we found this diversity effect in *A. elatius* and *D. glomerata*, which are highly productive species in the dominance experiment, while the two low‐productive species, *P. trivialis* and *A. pratensis*, showed no or the opposite effect (plants from low‐diversity communities had higher performance). This indicates that the abundance of a species could play an important role for eco‐evolutionary feedbacks, as higher abundance increases the probability of the presence of beneficial genotypes (higher defense against pathogens or higher interaction with mutualists) and/or selection for greater niche complementarity, which enables a stable population over time. This result highlights that especially the dominant, high‐productive species contribute to the phenomenon of the strengthening positive diversity–productivity relationship.

Another factor can be maternal effects: The phenotype of plants is dependent on the conditions which the mother has experienced (Herman & Sultan, 2011; De Long et al., 2019; De Long et al., 2020). This happens through the provisioning of maternally derived substances, such as nutrients, defensive chemicals, or hormones to the seeds (Herman & Sultan, 2011). Moreover, heritable (epigenetic) alterations may influence trait expression and performance of offspring selected in environments of different diversity. In our study, *P. trivialis* and to some extent also *A. pratensis* (both low‐productive species) showed higher performance (and survival) when originated from low‐diversity communities. This can be explained by at least two effects. First, lower performance of plants from high‐diversity communities could be caused by maternal effects and reduced seed provisioning. The abundance of *P. trivialis* and *A. pratensis* in the origin plant communities strongly decreased with increasing species richness, probably due to higher competition by tall‐growing plant species and thus deprivation of light, which in turn could lower the quality of the seeds and subsequently the performance of the offspring (Herman & Sultan, 2011). Second, plants from low‐diversity communities could be better adapted to higher light availability but also dryer microclimate due to lower shading in the less productive low‐diversity plant communities (Lorentzen et al., 2008; Wright et al., 2017), which could increase the performance of offspring from low‐diversity communities during the drought.

Contrary to our expectations, offspring of plants from low‐ and high‐diversity plant communities did not differ in leaf damage. In general, we found a very low herbivory rate, but a high infestation by rust, which is most likely explainable by the increasing mean air temperature over the last 12 years and the low precipitation in 2018 and 2019 in the Jena Experiment (Cappelli et al., 2020; Prather et al., 2020; Rai et al., 2018). The identified crown rust *Puccinia coronata* was the main driver of leaf damage of grasses, which is in line with previous studies in the Jena Experiment (Roscher et al., 2007; Rottstock et al., 2014). Despite the high infestation, plant history had no influence on leaf damage, indicating that plants selected in low‐ and high‐diversity communities did not differ in their ability to defend against leaf pathogens. This is in contrast to previous studies (Hahl et al., 2018; Roscher, Schumacher, Foitzik, et al., 2007), which showed that fungal infestation depends on intraspecific differences within the host species. It is likely caused by the different designs of the above‐mentioned studies and our study: We investigated plant species, which had always the same density within one plot (15 individuals), while in the other studies, the plants were growing in plant communities differing in plant species density. Probably, fungal infestation depends to a large extent on weather conditions, the actual growth environment, and neighboring plants, and to a lower extent on differences in the plant history.

In line with the leaf damage results, we found no effects of plant history on the expression of leaf traits (except higher leaf greenness of *P. trivialis* in home than in away‐same soil in May 2019). This suggests that phenotypic differences in leaf trait expression in response to community diversity, which was shown in several studies (Bachmann et al., 2018; Gubsch et al., 2011), were not induced by eco‐evolutionary feedbacks, but only by phenotypic plasticity in response to diversity and composition of the actual community, which is in line with a study by Miehe‐Steier et al. (2015). Nevertheless, our findings contrast previous studies (van Moorsel, Hahl, et al., 2018; van Moorsel et al., 2018; Zuppinger‐Dingley et al., 2014) showing different phenotypic trait expression of plants selected at low and high diversity. A potential explanation for the different results is that we transplanted the phytometers as single individuals in vegetation‐free field plots (in Miehe‐Steier et al. (2015) plants were grown as single individuals in pots), while plants in studies with contrasting results were grown as mixed communities in pots. Competition among the plant individuals is likely a more important driver for phenotypic plasticity of leaf morphological traits than plant history.

Hypothesis 3 Phytometers transplanted in high‐diversity soil show higher performance than phytometers in low‐diversity soil.

We found little effect of the actual soil environment on the performance of the phytometers. This is explainable by the following fact: Phytometers originated from high‐diversity communities produced more biomass in high‐diversity soil (“home” species richness) than in soil of low‐diversity communities (“away” species richness); however, this positive diversity effect is compensated by higher performance of plants from low‐diversity communities in low‐diversity soil (“home” species richness) than in soil of high‐diversity communities (“away” species richness; see Figure S5). This again demonstrates the "home advantage" and that plants evolved differently depending on whether they were growing at high or low diversity. One exception is *P. trivialis*, where both phytometers from low‐diversity and high‐diversity communities benefitted from the transplantation in soil of high‐diversity communities (in spring 2019). Due to the fact that *P. trivialis* is a species with a generally low productivity in the dominance experiment, this could be the reason for the different response compared with the other species.

## 5 CONCLUSION

Our study emphasizes that plant diversity loss and the associated changes in plant–soil feedbacks influence the micro‐evolutionary processes, which are measurable in the plant phenotype. These eco‐evolutionary feedbacks likely result in a higher investment of plants selected at low diversity into belowground defense, while plants selected at high diversity are able to invest more into growth, suggesting a growth defense trade‐off. Herewith, we provide first empiric evidence that not only changes in the abiotic and biotic conditions over time, but also eco‐evolutionary feedbacks contribute to the phenomenon of the strengthening positive diversity–productivity relationship. To confirm our results, we encourage future research that focuses on the long‐term performance of plants selected at low and high diversity and disentangle effects of soil biota from effects of soil properties determining the performance of these plants in home and away soil.

## CONFLICT OF INTEREST

The authors declare no conflict of interest.

## AUTHOR CONTRIBUTIONS

**Peter Dietrich:** Conceptualization (equal); Data curation (lead); Formal analysis (lead); Funding acquisition (equal); Investigation (lead); Writing‐original draft (lead). **Nico Eisenhauer:** Funding acquisition (equal); Supervision (supporting); Writing‐review & editing (equal). **Peter Otto:** Investigation (supporting); Writing‐review & editing (equal). **Christiane Roscher:** Conceptualization (equal); Investigation (supporting); Supervision (lead); Writing‐original draft (supporting); Writing‐review & editing (equal).

## Supporting information

Supplementary Material

## Data Availability

The data reported in this paper have been deposited in Dryad, which can be publicly accessed at https://doi.org/10.5061/dryad.qrfj6q5fn.
