# Supplementary material for: Plant history and soil history jointly influence the selection environment for plant species in a long‐term grassland biodiversity experiment
Source: Ecol Evol. 2021 May 11;11(12):8156–69. doi: 10.1002/ece3.7647 (PMC8216899; doi:10.1002/ece3.7647)

**Supplementary material**

**Article:** Plant history and soil history jointly influence the selection environment for plant species in a long-term grassland biodiversity experiment

**Authors:** Peter Dietrich*, Nico Eisenhauer, Peter Otto, Christiane Roscher

*corresponding author: peter.dietrich@idiv.de

**Tables**

**Table S1** Summary of linear mixed-effect model analyses testing the effects of the initial number of shoots, species identity (Species ID), origin species richness (Origin Sr; two- or six-species community), actual species richness (Actual Sr; two- or six-species community), soil treatment (away-different, away-same, home), season (June, September), and their interactions on number of shoots in 2018 with four species and three species (without *Poa trivialis*), respectively. Shown are degrees of freedom (Df), Chi^2^ and p-values (P). Significant factors and interactions are given in bold, marginally significant effects given in italics.

|  | With initial number of shoots and four species | | | Four species | | | Three species | | |
| --- | --- | --- | --- | --- | --- | --- | --- | --- | --- |
|  | Df | Chi^2^ | P | Df | Chi^2^ | P | Df | Chi^2^ | P |
| Initial number of shoots | 5 | <0.01 | 0.958 |  |  |  |  |  |  |
| Species ID | 8 | 26.59 | **<0.001** | 7 | 25.34 | **<0.001** | 6 | 45.97 | **<0.001** |
| Origin Sr | 9 | 0.01 | 0.926 | 8 | <0.01 | 0.960 | 7 | 0.12 | 0.725 |
| Actual Sr | 10 | 0.15 | 0.694 | 9 | 0.07 | 0.793 | 8 | 0.29 | 0.589 |
| Soil treatment | 12 | 0.92 | 0.630 | 11 | 0.71 | 0.702 | 10 | 1.20 | 0.549 |
| Season | 13 | 282.79 | **<0.001** | 12 | 281.88 | **<0.001** | 11 | 275.93 | **<0.001** |
| Species ID x origin Sr | 16 | 1.62 | 0.655 | 15 | 1.83 | 0.608 | 13 | 2.61 | 0.272 |
| Species ID x actual Sr | 19 | 5.44 | 0.142 | 18 | 5.05 | 0.168 | 15 | 6.03 | **0.049** |
| Species ID x soil treatment | 25 | 1.28 | 0.973 | 24 | 1.07 | 0.983 | 19 | 3.47 | 0.483 |
| Species ID x season | 28 | 284.47 | **<0.001** | 27 | 282.33 | **<0.001** | 21 | 170.09 | **<0.001** |
| Origin Sr x season | 29 | 2.07 | 0.150 | 28 | 2.07 | 0.151 | 22 | 0.08 | 0.781 |
| Actual Sr x season | 30 | 1.54 | 0.215 | 29 | 1.39 | 0.238 | 23 | 0.43 | 0.514 |
| Soil treatment x season | 32 | 1.58 | 0.454 | 31 | 1.60 | 0.450 | 25 | 2.38 | 0.304 |
| Species ID x origin Sr x season | 35 | 8.49 | **0.037** | 34 | 8.42 | **0.038** | 27 | 9.06 | **0.011** |
| Species ID x actual Sr x season | 38 | 5.36 | 0.147 | 37 | 5.00 | 0.172 | 29 | 8.48 | **0.014** |
| Species ID x soil treatment x season | 44 | 0.41 | 0.999 | 43 | 0.44 | 0.999 | 33 | 0.77 | 0.943 |

**Table S2** Summary of linear mixed-effect model analyses testing the effects of origin species richness (Origin Sr; two- or six-species community), actual species richness (Actual Sr; two- or six-species community) and soil treatment (away-different, away-same, home) on number of shoots in June and September 2018 for each of the used plant species, respectively. Shown are degrees of freedom (Df), Chi^2^ and p-values (P). Significant factors and interactions are given in bold, marginally significant effects given in italics.

|  | Number of shoots in June 2018 | | | | | | | | | | | |
| --- | --- | --- | --- | --- | --- | --- | --- | --- | --- | --- | --- | --- |
|  | *A. elatius* | | | *A. pratensis* | | | *D. glomerata* | | | *P. trivialis* | | |
|  | Df | Chi^2^ | P | Df | Chi^2^ | P | Df | Chi^2^ | P | Df | Chi^2^ | P |
| Origin Sr | 5 | 1.09 | 0.296 | 5 | 1.36 | 0.244 | 5 | 1.72 | 0.190 | 5 | 0.05 | 0.826 |
| Actual Sr | 6 | 8.55 | **0.003** | 6 | 0.08 | 0.774 | 6 | 0.27 | 0.606 | 6 | 2.42 | 0.120 |
| Soil treatment | 8 | 3.24 | 0.198 | 8 | 6.60 | **0.037** | 8 | 4.46 | 0.108 | 8 | 1.27 | 0.530 |
|  | Number of shoots in September 2018 | | | | | | | | | | | |
|  | *A. elatius* | | | *A. pratensis* | | | *D. glomerata* | | | *P. trivialis* | | |
|  | Df | Chi^2^ | P | Df | Chi^2^ | P | Df | Chi^2^ | P | Df | Chi^2^ | P |
| Origin Sr | 5 | 1.09 | 0.298 | 5 | 2.86 | *0.091* | 5 | 4.28 | **0.039** | 5 | 4.12 | **0.042** |
| Actual Sr | 6 | 0.43 | 0.513 | 6 | 2.55 | 0.111 | 6 | 4.01 | **0.045** | 6 | 1.26 | 0.262 |
| Soil treatment | 8 | 3.65 | 0.162 | 8 | 6.53 | **0.038** | 8 | 9.36 | **0.009** | 8 | 1.10 | 0.576 |

**Table S3** Summary of linear mixed-effect model analyses testing the effects of origin species richness (Origin Sr; two- or six-species community), actual species richness (Actual Sr; two- or six-species community) and soil treatment (away-different, away-same, home) on aboveground biomass production in May and August 2019 for each of the used plant species, respectively. Shown are degrees of freedom (Df), Chi^2^ and p-values (P). Significant effects are given in bold, marginally significant effects given in italics.

|  | Aboveground biomass production in May 2018 | | | | | | | | | | | |
| --- | --- | --- | --- | --- | --- | --- | --- | --- | --- | --- | --- | --- |
|  | *A. elatius* | | | *A. pratensis* | | | *D. glomerata* | | | *P. trivialis* | | |
|  | Df | Chi^2^ | P | Df | Chi^2^ | P | Df | Chi^2^ | P | Df | Chi^2^ | P |
| Origin Sr | 5 | 3.90 | **0.048** | 5 | 0.19 | 0.662 | 5 | 2.92 | *0.087* | 5 | 3.45 | *0.063* |
| Actual Sr | 6 | 1.91 | 0.167 | 6 | 0.65 | 0.422 | 6 | 0.45 | 0.502 | 6 | 4.28 | **0.039** |
| Soil treatment | 8 | 1.79 | 0.409 | 8 | 3.17 | 0.205 | 8 | 3.97 | 0.137 | 8 | 0.58 | 0.749 |
|  | Aboveground biomass production in August 2019 | | | | | | | | | | | |
|  | *A. elatius* | | | *A. pratensis* | | | *D. glomerata* | | | *P. trivialis* | | |
|  | Df | Chi^2^ | P | Df | Chi^2^ | P | Df | Chi^2^ | P | Df | Chi^2^ | P |
| Origin Sr | 5 | 4.20 | **0.041** | 5 | 0.00 | 0.954 | 5 | 5.04 | **0.025** | NA | NA | NA |
| Actual Sr | 6 | 1.15 | 0.284 | 6 | 0.03 | 0.868 | 6 | 0.30 | 0.586 | NA | NA | NA |
| Soil treatment | 8 | 1.23 | 0.540 | 8 | 0.84 | 0.656 | 8 | 3.83 | 0.148 | NA | NA | NA |

**Table S4** Summary of linear mixed-effect model analyses testing the effects of species identity (Species ID), origin species richness (Origin Sr; two- or six-species community), actual species richness (Actual Sr; two- or six-species community), soil treatment (away-different, away-same, home), and their interactions on leaf greenness in May and August 2019 (in May with and without *P. trivialis*, because of 45% mortality; in August without *P. trivialis*, because of 100% mortality). Shown are degrees of freedom (Df), Chi^2^ and p-values (P). Significant factors and interactions are given in bold, marginally significant effects given in italics.

|  | May 2019 | | | | | | August 2019 | | |
| --- | --- | --- | --- | --- | --- | --- | --- | --- | --- |
|  | four species | | | three species | | | four species | | |
|  | Df | Chi^2^ | P | Df | Chi^2^ | P | Df | Chi^2^ | P |
| Species ID | 7 | 74.49 | **<0.001** | 6 | 31.30 | **<0.001** | 6 | 16.22 | **<0.001** |
| Origin Sr | 8 | 0.49 | 0.486 | 7 | 0.59 | 0.442 | 7 | 0.02 | 0.881 |
| Actual Sr | 9 | 1.31 | 0.252 | 8 | 0.33 | 0.564 | 8 | 0.51 | 0.475 |
| Soil treatment | 11 | 2.34 | 0.310 | 10 | 1.10 | 0.578 | 10 | 1.55 | 0.461 |
| Species ID x origin Sr | 14 | 7.19 | *0.066* | 12 | 1.04 | 0.595 | 12 | 1.20 | 0.550 |
| Species ID x actual Sr | 17 | 2.52 | 0.472 | 14 | 2.27 | 0.321 | 14 | 1.54 | 0.463 |
| Species ID x soil treatment | 23 | 28.62 | **<0.001** | 18 | 2.53 | 0.639 | 18 | 2.17 | 0.704 |

**Table S5** Summary of linear mixed-effect model analyses testing the effects of species identity (Species ID), origin species richness (Origin Sr; two- or six-species community), actual species richness (Actual Sr; two- or six-species community), soil treatment (away-different, away-same, home), and their interactions on specific leaf area and leaf infestation in August 2019 (without *P. trivialis*, because of 100% mortality). Shown are degrees of freedom (Df), Chi^2^ and p-values (P). Significant factors and interactions are given in bold, marginally significant effects given in italics.

|  | Specific leaf area | | | Leaf infestation | | |
| --- | --- | --- | --- | --- | --- | --- |
|  | Df | Chi^2^ | P | Df | Chi^2^ | P |
| Species ID | 6 | 13.85 | **0.001** | 6 | 18.11 | **<0.001** |
| Origin Sr | 7 | 0.14 | 0.711 | 7 | 0.00 | 0.992 |
| Actual Sr | 8 | 0.16 | 0.687 | 8 | 0.62 | 0.429 |
| Soil treatment | 10 | 1.11 | 0.574 | 10 | 4.24 | 0.120 |
| Species ID x origin Sr | 12 | 1.61 | 0.447 | 12 | 4.38 | 0.112 |
| Species ID x actual Sr | 14 | 0.61 | 0.739 | 14 | 0.36 | 0.834 |
| Species ID x soil treatment | 18 | 1.98 | 0.740 | 18 | 2.82 | 0.589 |

**Table S6** Summary of linear mixed-effect model analyses testing the effects of origin species richness (Origin Sr; two- or six-species community), actual species richness (Actual Sr; two- or six-species community) and soil treatment (away-different, away-same, home) on leaf greenness in May for each of the used plant species, respectively. Shown are degrees of freedom (Df), Chi^2^ and p-values (P). Significant factors and interactions are given in bold, marginally significant effects given in italics.

|  | *A. elatius* | | | *A. pratensis* | | | *D. glomerata* | | | *P. trivialis* | | |
| --- | --- | --- | --- | --- | --- | --- | --- | --- | --- | --- | --- | --- |
|  | Df | Chi^2^ | P | Df | Chi^2^ | P | Df | Chi^2^ | P | Df | Chi^2^ | P |
| Origin Sr | 5 | 1.44 | 0.230 | 5 | 0.18 | 0.675 | 5 | 0.22 | 0.635 | 5 | 2.86 | *0.091* |
| Actual Sr | 6 | 2.22 | 0.136 | 6 | 0.02 | 0.895 | 6 | 0.21 | 0.651 | 6 | 1.05 | 0.305 |
| Soil treatment | 8 | 4.04 | 0.132 | 8 | 0.03 | 0.987 | 8 | 0.49 | 0.784 | 8 | 10.47 | **0.005** |

**Table S7** Summary of linear mixed-effect model analyses testing the effects of species identity (Species ID), biomass production, and their interaction on number of inflorescences (No of inflorescences) in May 2019 as well as linear mixed-effect model analyses for each species, respectively. Shown are degrees of freedom (Df), Chi^2^ and p-values (P). Significant factors and interactions are given in bold, marginally significant effects given in italics.

|  | No of inflorescences | | |
| --- | --- | --- | --- |
|  | Df | Chi^2^ | Df |
| Four species |  |  |  |
| Species ID | 7 | 33.57 | **<0.001** |
| Biomass production | 8 | 186.10 | **<0.001** |
| Species x biomass prod. | 12 | 47.20 | **<0.001** |
| *A. elatius* |  |  |  |
| Biomass production | 5 | 64.75 | **<0.001** |
| *A. pratensis* |  |  |  |
| Biomass production | 5 | 25.86 | **<0.001** |
| *D. glomerata* |  |  |  |
| Biomass production | 5 | 66.19 | **<0.001** |
| *P. trivialis* |  |  |  |
| Biomass production | 5 | 63.75 | **<0.001** |

**Figures**

**Figure S1** Air temperature and precipitation from 2007 to 2019 for the full year (temperature: a; precipitation: c) and for the summer months (June, July, August; temperature: b; precipitation: d). Each bar represents the value for one year. Gray lines indicate significant increase or decrease over time derived from a simple linear regression. *R* indicates the Pearson’s correlation coefficient and *p* the associated level of significance. Data were recorded by the weather station Jena-Saaleaue of the Max-Planck-Institute Jena.


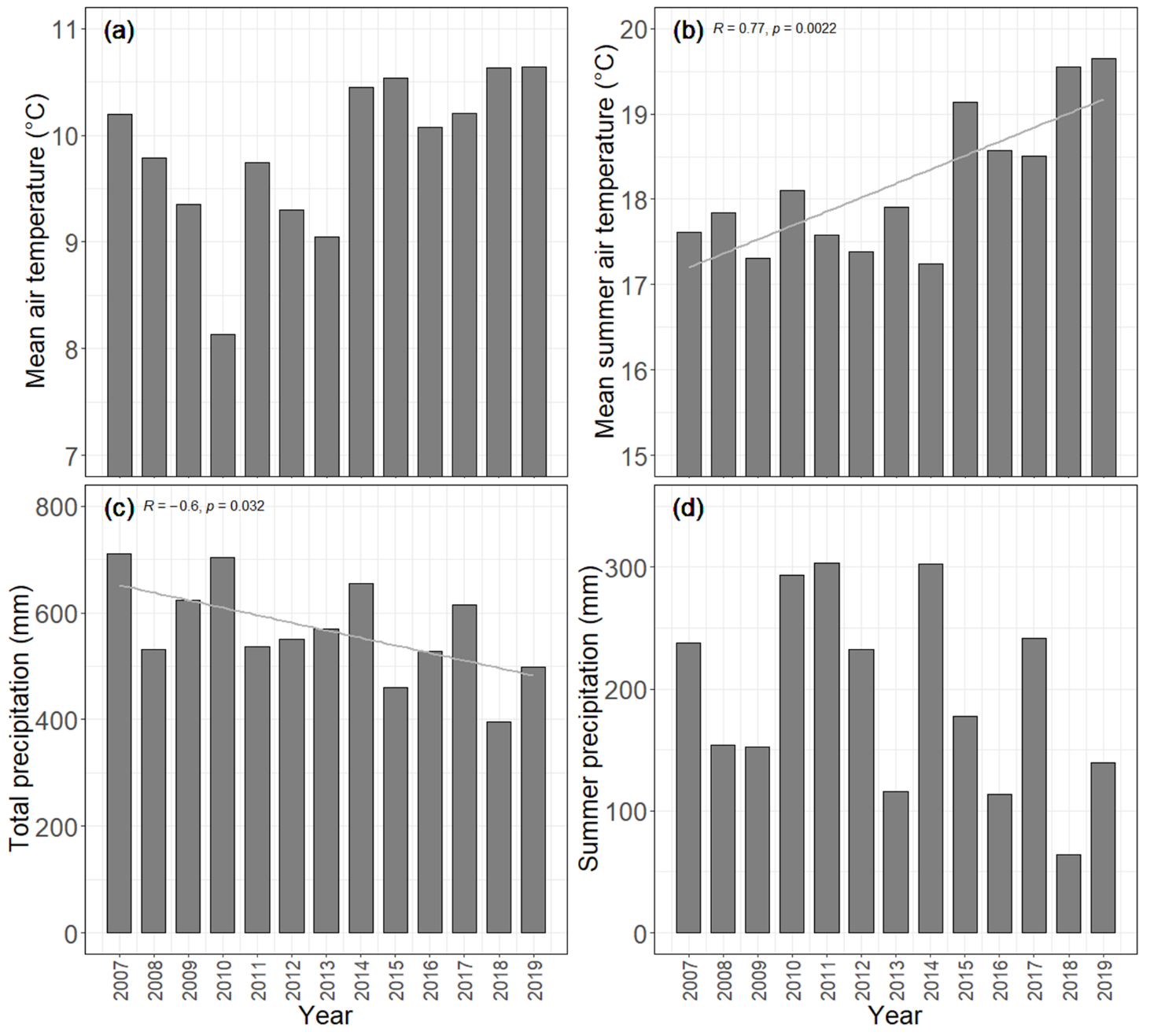


**Figure S2** Relative biomass production (percentage of community biomass) of the four used grass species in the research plots in 2017. For biomass harvest, a 0.2 x 0.5 m sample area per plot was randomly chosen and plants were cut five cm above ground, in June 2017. When only a few individuals were present, the sample area was doubled. Biomass was sorted by species, dried at 70°C for 48 h and weighted. Biomass production per plot was extrapolated to one square meter. Bars show mean values (± 1 SE).


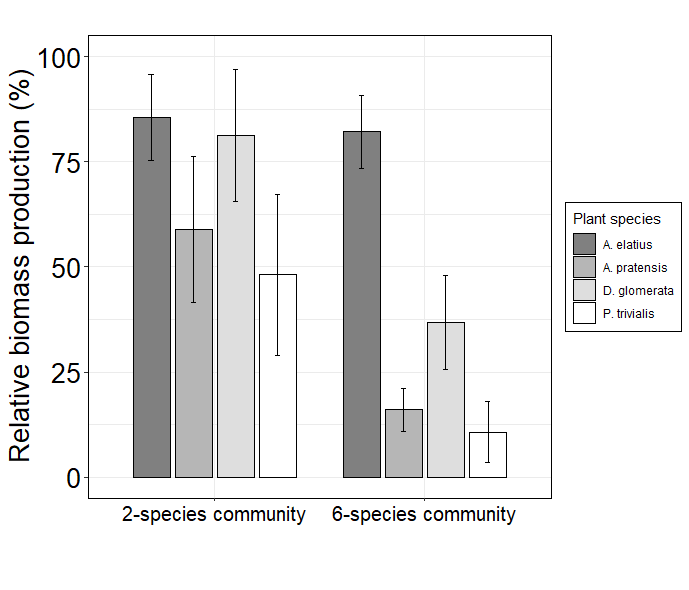


**Figure S3** Leaf greenness of phytometers in away-different, away-same and home soil for each species, in May 2019. Bars show mean values (± 1 SE); stars above bars indicate significant (p < 0.05) differences.


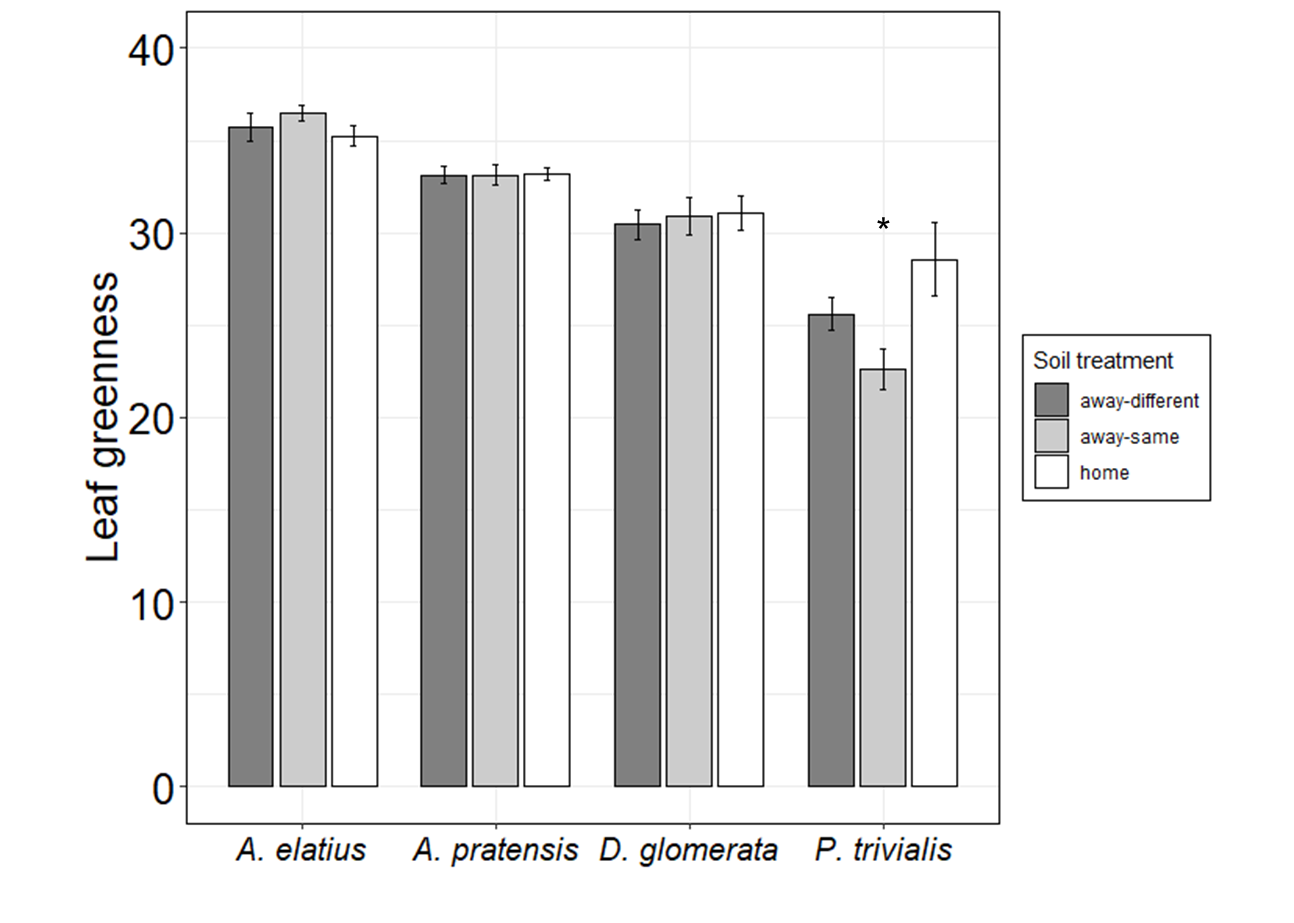


**Figure S4** Number of inflorescences plotted against aboveground biomass production for three species (without *P. trivialis*; a) and for four species (with *P. trivialis*: b) in May 2019. The solid line indicates positive relationship derived from a simple linear regression. Each dot represents one phytometer. *R* indicates the Pearson’s correlation coefficient and *p* the associated level of significance.


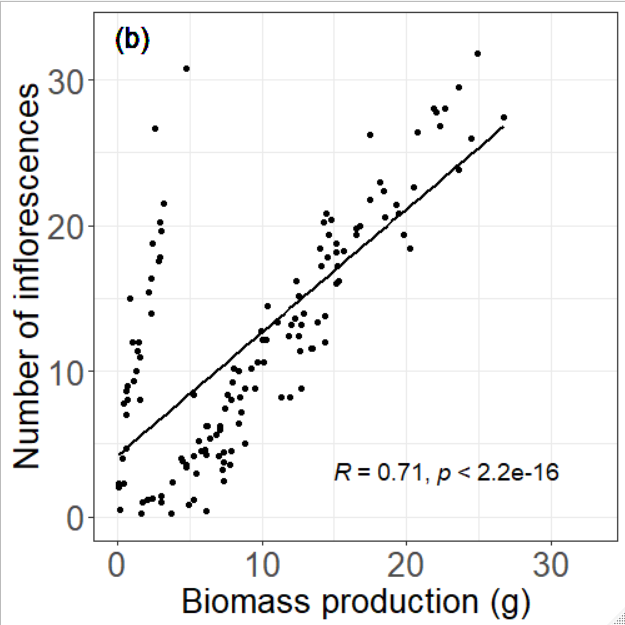

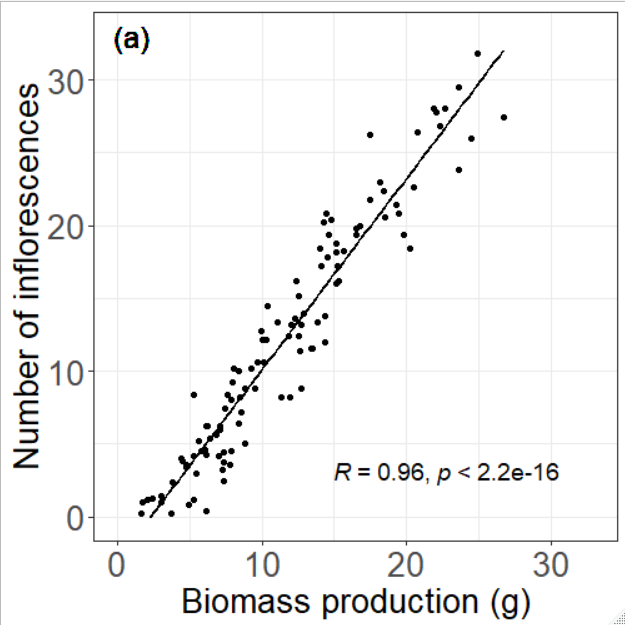


**Figure S5** Aboveground biomass production of phytometers (without *Poa trivialis*), when transplanted into soil of two- and six-species communities (actual species richness) in May (a, b) and August (c, d) 2019. Shown are total biomass production (a, c) and biomass production separated for plants originated from two- and six-species communities (separated by origin species richness; b, d). Bars show mean values (± 1 SE) and lines indicate biomass production changes, when plants were grown in home species richness soil (same species richness) vs. in away species richness soil (different species richness).


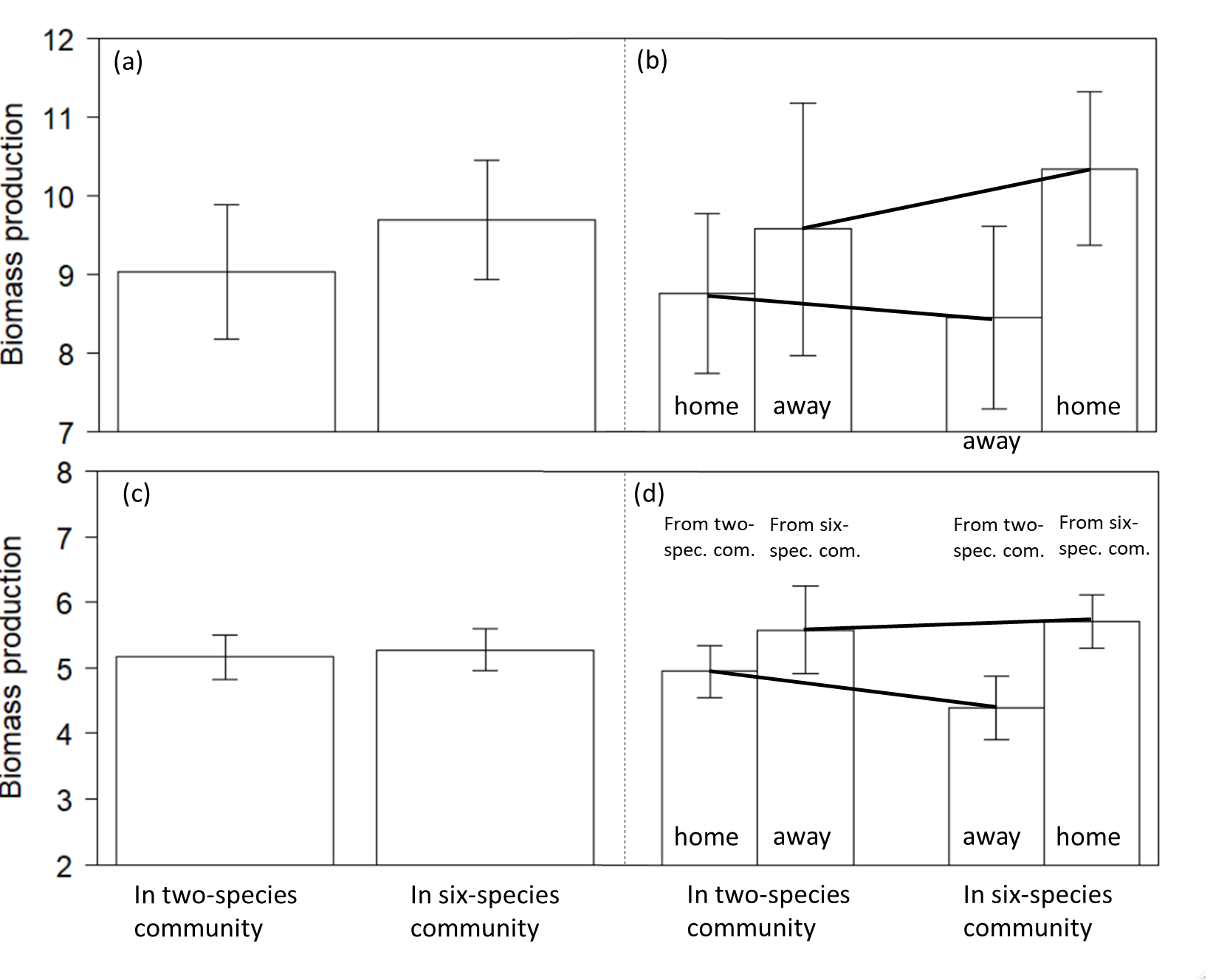

Supplement: Supplementary file 1 — Supplementary Material [file ECE3-11-8156-s001.docx]
